# Effect of Anagliptin and Sitagliptin on Low-Density Lipoprotein Cholesterol in Type 2 Diabetic Patients with Dyslipidemia and Cardiovascular Risk: Rationale and Study Design of the REASON Trial

**DOI:** 10.1007/s10557-018-6776-z

**Published:** 2018-02-12

**Authors:** Shinichiro Ueda, Michio Shimabukuro, Osamu Arasaki, Koichi Node, Takashi Nomiyama, Takeshi Morimoto

**Affiliations:** 1 Department of Clinical Pharmacology and Therapeutics, University of the Ryukyus, 207 Uehara, Nishihara, Okinawa 903-0215 Japan; 2 Department of Diabetes, Endocrinology and Metabolism, Fukushima Medical University, Fukushima, Japan; 3 Department of Cardiology, Tomishiro Central Hospital, Okinawa, Japan; 4 Department of Cardiovascular Medicine, Saga University, Saga, Japan; 5 Department of Endocrinology and Diabetes Mellitus, Fukuoka University, Fukuoka, Japan; 6 Department of Clinical Epidemiology, Hyogo College of Medicine, Nishinomiya, Japan

**Keywords:** DPP-4 inhibitors, Low-density lipoprotein cholesterol, Type 2 diabetic patients, Dyslipidemia, Cardiovascular risk

## Abstract

**Background:**

Reduction of low-density lipoprotein cholesterol (LDL-C) is important for patients with a high risk for atherosclerotic events, such as patients with diabetes and other risk factors. Anagliptin was reported to reduce LDL-C for 12 weeks in phase III trials regardless of the use of statins, but it is uncertain whether this effect is common to other dipeptidylpeptidase-4 (DPP-4) inhibitors.

**Methods:**

A multicenter, randomized, open-label, parallel-group trial was conducted to confirm the superiority of anagliptin to sitagliptin in terms of the primary endpoint of reduction of LDL-C for 52 weeks in patients with type 2 diabetes and atherosclerotic vascular lesions, as well as the non-inferiority of anagliptin to sitagliptin in terms of change in hemoglobin A1c (HbA1c). Patients are randomly assigned to receive anagliptin or sitagliptin at a ratio of 1:1, with those in the anagliptin group receiving anagliptin 100 mg orally twice per day and those in the sitagliptin group receiving sitagliptin 50 mg orally once per day. During the trial period, hypoglycemic agents and anti-dyslipidemia drugs should not be added and neither should their dosages be changed. A total sample size of 300 was estimated to provide a power of 0.8 with a two-sided alpha of 0.05 for LDL-C, considering a 30% dropout rate. Pre-specified factors for subgroup analyses are HbA1c, use of DPP-4 inhibitors, sex, body mass index, LDL-C, age, and the presence of treatment for existing ischemic heart disease.

**Discussion:**

If anagliptin were to be shown to reduce LDL-C in patients with type 2 diabetes and atherosclerotic vascular lesions despite pre-existing statin treatment, more intensive cholesterol management would be appropriate.

**Trial Registration:**

Clinicaltrials.gov NCT02330406

**Electronic supplementary material:**

The online version of this article (10.1007/s10557-018-6776-z) contains supplementary material, which is available to authorized users.

## Background

Diabetes mellitus is one of the strongest risk factors for atherosclerotic diseases [1]. In Europe and the USA, myocardial infarction is the direct cause of death in 40–50% of patients with diabetes, and the number of patients with diabetes that die as a direct result of ischemic heart disease is reportedly increasing [2]. In a Finnish study, the rate of incipient myocardial infarction among patients with diabetes was roughly equal to the rate of recurrent myocardial infarction among non-diabetic patients; furthermore, patients with diabetes and prior myocardial infarction demonstrated a greatly increased incidence of myocardial infarction [3]. Although the application of such results to the Japanese population is not straightforward, existing atherosclerotic vascular lesions and diabetes mellitus are undoubtedly major risk factors for further fatal vascular diseases. Therefore, multifactorial intervention, including control of blood glucose, blood pressure, and lipid levels, is crucial for the prevention of vascular disease [4].

Dipeptidylpeptidase-4 (DPP-4) inhibitors enhance the action of glucagon-like peptide 1 (GLP-1), the most physiologically important incretin, and they consequently show various actions, such as the stimulation of glucose-dependent insulin secretion, inhibition of glucagon secretion, inhibition of gastric emptying, and appetite regulation [5]. DPP-4 inhibitors have become standard drugs to improve hemoglobin A1c (HbA1c) levels in patients with diabetes, though improvement of cardiovascular outcomes by adding DPP-4 inhibitors to usual care in diabetic patients with established cardiovascular diseases has not yet been shown [6–8]. Anagliptin is unique among the DPP-4 inhibitors because it was reported to reduce low-density lipoprotein cholesterol (LDL-C) by 9.5 mg/dL over 12 weeks [9] in phase III trials regardless of the use of statins. This effect of anagliptin may not be a “class effect” of DPP-4 inhibitors, because there has been experimental evidence to suggest that anagliptin inhibits the absorption of cholesterol in the small intestine and cholesterol synthesis in the liver, which presumably are involved in the LDL-C-mediated reduction by anagliptin [10].

This LDL-C-lowering effect of anagliptin is particularly relevant to diabetic patients with high cardiovascular risk, but such patients are likely to be excluded from clinical trials for regulatory approval. The aim of the present study is, therefore, to investigate the comparative effects of anagliptin and sitagliptin, which was most frequently prescribed in Japan, on LDL-C in patients with type 2 diabetes with dyslipidemia and atherosclerotic vascular lesions.

## Methods

### Trial Design

This is a multicenter, randomized, open-label, parallel-group trial to determine the effectiveness of anagliptin versus sitagliptin on reduction in LDL-C in patients with type 2 diabetes and existing atherosclerotic vascular lesions. Patients with type 2 diabetes and existing atherosclerotic vascular lesions under treatment of statin therapy are included. The eligibility criteria are shown in Table 1.

**Table 1:** Patient eligibility criteria

Inclusion criteria	Patients who fulfilled all of the following criteria were included
1	High-risk (*) patients with type 2 diabetes who are undergoing diet therapy/exercise therapy or are using other hypoglycemic agents in conjunction with diet therapy/exercise therapy
2	Patients who have been using statins for ≥8 weeks
3	Patients with LDL-C ≥ 100 mg/gL in ≥ 1 of their previous three measurements after the use of statins
4	Patients with HbA1c ≥ 6.0% and < 10.5% (if the investigational drug is added on, HbA1c ≥ 7.0% and < 10.5%)
5	Patients aged ≥ 20 years at the time of consent
6	Patients who provide written consent to participate in the trial of their own free will based on a sufficient understanding of the trial following an adequate explanation
Exclusion criteria	Patients who met any of the following criteria were excluded
1	Patients with type 1 diabetes
2	Patients with TG ≥ 400 mg/dL in a past fasting blood sample
3	Women who are pregnant, potentially pregnant, or lactating
4	Patients with severe infections, who are scheduled to undergo/have just undergone surgery, or who have serious trauma
5	Patients with a serum creatinine level ≥ 2.4 mg/dL for men or ≥ 2.0 mg/dL for women
6	Patients using GLP-1 receptor agonists
7	Patients considered ineligible for any other reason by a study investigator
* High-risk	Defined as the fulfillment of any one of the following criteria
1	Stenotic lesions or plaques of ≥ 25% of the arterial diameter in past coronary angiography or CT
2	Coronary artery calcification in past coronary CT
3	Past history of acute coronary syndrome
4	Past history of PCI or CABG
5	Past history of stroke (ischemic cerebral infarction or cerebral hemorrhage)
6	Past history of TIA
7	Past history of peripheral artery disease (including aortic lesions)
8	Past ankle-brachial index ≤ 0.9
9	Presence of carotid artery plaque (including max IMT ≥ 1.1 mm) in past carotid duplex

CABG: coronary artery bypass surgery; CT: computed tomography; GLP-1: glucagon-like peptide-1; HbA1c: hemoglobin A1c; IMT: intima-media thickness; LDL-C: low-density lipoprotein cholesterol; TG: triglyceride; PCI: percutaneous coronary intervention; TIA: transient ischemic attack

Registration, randomization, and data collection are performed using an electronic data capture (EDC) system. Randomization is performed centrally through the EDC system with a stochastic minimization algorithm to balance treatment assignment within and across hospitals, HbA1c (≥ 8.0%, < 8.0%, use of DPP-4 inhibitors prior to trial registration, sex, body mass index (BMI) (≥ 25 kg/m^2^, < 25 kg/m^2^), and LDL-C (≥ 130 mg/dL, < 130 mg/dL).

### Trial Oversight

The principal investigator, co-principal investigator, and members of the steering committee designed and conducted this study in accordance with the Ethical Guidelines for Medical and Health Research Involving Human Subjects in Japan (Table 2). Protocol and consent forms were approved by the institutional review boards at the University of the Ryukyus (No. 731) and each participating center (Table 3) to which important protocol modification and safety information including serious adverse events in this trial are reported. All patients or their legally authorized representatives provide written, informed consent before randomization with investigators. All centers are regularly monitored by steering committee representatives, and the trial is monitored by an independent data and safety monitoring board (Table 2). Auditing this trial is conducted by the audit department of the University of the Ryukyus independently. This trial was registered at Clinicaltrials.gov (NCT02330406).

**Table 2:** Study oversight

Role of study	Name	Institution
Principal investigator Steering Committee	Shinichiro Ueda, MD, PhD	Department of Pharmacology and Therapeutics, University of the Ryukyus
Co-principal investigator Steering Committee	Takeshi Morimoto, MD, PhD, MPH	Department of Clinical Epidemiology, Hyogo College of Medicine
Steering Committee	Osamu Arasaki, MD	Department of Cardiology, Tomishiro Central Hospital
Steering Committee	Koichi Node, MD, PhD	Department of Cardiovascular Medicine, Saga University
Steering Committee	Michio Shimabukuro, MD, PhD	Department of Diabetes, Endocrinology and Metabolism, Fukushima Medical University
Steering Committee	Takashi Nomiyama, MD, PhD	Department of Endocrinology and Diabetes Mellitus, Fukuoka university,
Event Adjudication Committee	Akihiro Tokushige, MD, PhD	Department of Pharmacology and Therapeutics, University of the Ryukyus
Event Adjudication Committee	Masahiro Natsuaki, MD, PhD	Department of Cardiovascular Medicine, Saga University
Event Adjudication Committee	Tomohiro Asahi, MD, PhD	Department of Cardiology, Naha City Hospital
Data Safety Monitoring Board	Keijiro Saku, MD, PhD	General Medical Research Center, Fukuoka University
Data Safety Monitoring Board	Tetsunori Saikawa, MD, PhD	Okubo Hospital
Data Safety Monitoring Board	Kohei Kaku, MD, PhD	Department of General Internal Medicine 1, Kawasaki Medical School
Study statistician	Mio Sakuma, MD, PhD, MPH	Institute for Clinical Effectiveness
Study statistician	Takeshi Morimoto, MD, PhD, MPH	Department of Clinical Epidemiology, Hyogo College of Medicine
Study secretariat	–	Department of Pharmacology and Therapeutics, University of the Ryukyus
Project management	–	Institute for Clinical Effectiveness
Data management	–	Institute for Clinical Effectiveness
Study advisor	Hisao Ogawa, MD, PhD	National Cerebral and Cardiovascular Center

**Table 3:** Participating centers and investigators

Center	Investigators
Hokko Memorial Clinic	Ichiro Sakuma, MD, PhD
Tomishiro Central Hospital	Osamu Arasaki, MD
Sunagawa Medical Clinic	Hiroshi Sunagawa, MD
Imari Arita Kyoritsu Hospital	Kazuo Matsunaga, MD, PhD
Oohama Daiichi Hospital	Ryu Takahashi, MD, PhD
Chibana Clinic	Isao Shiroma, MD
Northern Okinawa Cardiovascular Center	Kinya Ashida, MD
Baba Memorial Hospital	Hajime Yamashita, MD, PhD
JR Hiroshima Hospital	Hiroki Teragawa, MD, PhD
Kusatsu General Hospital	Atsuyuki Wada, MD, PhD
Shonan Hospital	Ken Nakachi, MD, PhD
Fukuoka University Hospital	Takashi Nomiyama, MD, PhD
Saiseikai Fukuoka General Hospital	Toru Kubota, MD, PhD
Urasoe General Hospital	Hiroki Uehara, MD
Naha City Hospital	Tomohiro Asahi, MD, PhD
Kokura Memorial Hospital	Takashi Morinaga, MD
Tokushima University Hospital	Masataka Sata, MD, PhD

### Trial Intervention

**Fig. 1:**
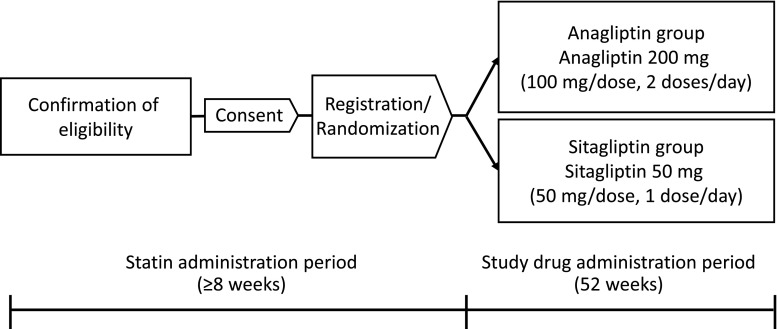
Study design. After the statin administration period and informed consent is obtained, eligible patients are randomly allocated to the anagliptin group who receive twice a day anagliptin at 200 mg per day and to the sitagliptin group who receive once a day sitagliptin at 50 mg per day for 52 weeks, the study drug treatment period

Patients randomly receive anagliptin or sitagliptin at a ratio of 1:1 (Fig. 1). Treatment assignment is not concealed from participants or treating physicians. Patients in the anagliptin group are given anagliptin 100 mg orally twice per day for 52 weeks. If the effects are insufficient, the dose can be increased to 200 mg orally twice per day. Patients in the sitagliptin group are given sitagliptin 50 mg orally once per day for 52 weeks. If the effects are insufficient, the dose can be increased to 100 mg per day. If the patients are using anti-diabetic drugs other than DPP-4 inhibitors at the start of the trial, the study drug is administered concomitantly, and such anti-diabetic drugs are not to be replaced.

During the trial period, hypoglycemic agents and anti-dyslipidemia drugs (statins, ezetimibe, anion exchange resins, fibrates, and eicosapentaenoic acid (EPA)) are not to be added, and their dosages are not to be changed. A change in dose of insulin is not considered a change in hypoglycemic agents. Other therapy is determined by the physician in charge.

Changes in the medication use as well as other drugs with possible effects on outcome are prohibited. Our clinical research coordinators regularly monitor participants and their physicians about adherence to the study medication/dose at every visit. If cross-over is found, the patients should be dropped out as per protocol.

### Measurements

Through clinical research coordinators or physicians in charge, information on patients is obtained. Clinical characteristics include age, sex, height, waist circumference, body weight, blood pressure, heart rate, past medical history, smoking status, alcohol consumption, drug allergies, and use of concomitant drugs. Blood pressure, heart rate, and compliance with study drug are monitored at 12, 24, 36, and 52 weeks. Height, waist circumference, and body weight were also measured at 52 weeks.

Blood glucose level, red blood cell count, white blood cell count, hemoglobin, hematocrit, platelet count, AST, ALT, γGTP, CK, BUN, and creatinine are measured locally at baseline, 12, 24, 36, and 52 weeks, and LDL-C, HbA1c, total cholesterol (TC), HDL-C, triglycerides (TG), apolipoprotein A1 (Apo-A1), apolipoprotein B (ApoB), apolipoprotein E (ApoE), glycoalbumin, insulin, 1,5-anhydro-D-glucitol (1,5-AG), and C-peptide are measured at the same time periods and analyzed at the core laboratory (SRL Inc., Tokyo, Japan). Small dense LDL, apolipoprotein B48 (ApoB48), high-sensitivity C-reactive protein (hsCRP), interleukin-6 (IL-6), campesterol, sitosterols, lathosterol, and high-molecular-weight adiponectin are measured at baseline and at 52 weeks and analyzed at the same core laboratory. Urine albumin and urine creatinine are also measured at baseline and 52 weeks and analyzed at the same core laboratory. Blood samples are obtained at fasting or at least 4 h after a meal. Follow-up is done at the hospital outpatient clinic or referring clinics.

Other substudies such as changes in the measurement of intima media thickness (IMT), detailed lipoprotein profiling, and fractions of fatty acids are included in the protocol. Substudies that are unplanned at this point should be conducted according to the decision of the Steering Committee. Additional serum samples for this purpose are stored.

### Endpoints

The primary endpoint is the change in LDL-C. LDL-C is calculated based on the Friedewald (F) equation. An important secondary endpoint is the change in HbA1c.

Secondary endpoints are changes in the following measurements: fasting blood glucose, fasting insulin, 1,5-AG, C-peptide, TC, TG, HDL-C, non-HDL-C, ApoA1, ApoB, ApoE, ApoB48, small dense LDL, hsCRP, IL-6, campesterol, sitosterols, lathosterol, high-molecular-weight adiponectin, estimated glomerular filtration rate (eGFR), and the urine albumin/creatinine ratio. Secondary endpoints also include progression, lack of change, or remission of microalbuminuria and macroalbuminuria.

Safety outcomes are death and any adverse events systematically reported for 52 weeks, including hypoglycemia, pancreatitis, pancreatic cancer, ileus, and cardiovascular and cerebrovascular events. Safety outcomes are defined in the protocol. Symptoms requiring hospital admission are included, according to the Japanese Ethical Guidelines.

### Data Management

The details of data management including data entry, coding, security, and storage, including any related processes to promote data quality, are described in the full protocol and more in the standard operation procedures issued by the data center (Institute of Clinical Effectiveness).

### Sample Size and Statistical Analysis

The present trial seeks to confirm that the anagliptin group is significantly superior to the sitagliptin group in terms of the primary endpoint of change in LDL-C after 52 weeks of treatment. Thus, the null hypothesis is that both anagliptin and sitagliptin groups demonstrate equal changes in LDL-C levels, while the alternative hypothesis is that the sitagliptin and anagliptin groups demonstrate different changes in LDL-C.

It was assumed that reductions in LDL-C after 52 weeks would be similar to a previous report that showed that anagliptin provided an LDL-C decrease of 9.5 mg/dL [9], whereas sitagliptin provided a decrease of 0.97 mg/dL [11], with standard deviation (SD) of 21.6 mg/dL for both treatments. With a two-sided alpha of 0.05 and power of 0.8, the sample size was calculated as 102 for each group. Considering a dropout rate of 30%, 300 was set as the total sample size.

This trial was also designed to confirm that the anagliptin group is non-inferior to the sitagliptin group in terms of the important secondary endpoint of the change in HbA1c. The non-inferiority margin of HbA1c was set to 0.3%, which is conventionally used for HbA1c. The null hypothesis is that the 97.5% upper one-sided confidence limit for [change in HbA1c in sitagliptin group − change in HbA1c in anagliptin group] ≥ 0.3, and the alternative hypothesis is that the 97.5% upper one-sided confidence limit for [change in HbA1c in sitagliptin group − change in HbA1c in anagliptin group] < 0.3.

A total sample size of 300 provides a power of 0.8 for HbA1c on the assumption that the SD for HbA1c in the anagliptin group based on a previous clinical trial is 0.91, and the non-inferiority margin is 0.3 with a one-sided alpha of 0.025.

The statistical analysis plan (SAP) will be specified before data analysis. The full analysis set (FAS) includes patients who received allocated treatment and provided assessable outcome data. The safety analysis set (SAS) includes patients who received allocated treatment at least once. The per protocol set (PPS) includes patients who received allocated treatment and for whom planned outcome data as per the protocol are available. The FAS is used for the primary endpoint, and the FAS and PPS are used for the important secondary and other secondary endpoints. The SAS is used for safety outcomes. Categorical variables are expressed as frequencies with percentages, and continuous variables are expressed as means with SDs or medians with interquartile ranges (IQRs). All analyses are conducted under the intention-to-treat principle.

Repeated ANOVA with mixed effect models for repeated measures (MMRM) will be developed to compare the anagliptin and sitagliptin groups in terms of change in LDL-C. In this model, treatment allocation and time are treated as fixed effects and include five variables used in balancing factors at randomization as covariates. The same model without covariates of stratification variables will also be developed for sensitivity analyses. The non-inferiority of the anagliptin group to the sitagliptin group in terms of HbA1c levels will be examined with a non-inferiority margin of 0.3%.

For the changes in other endpoints, a one-sample *t* test is used for comparisons of the baseline and follow-up values within the group, and two-sample *t* tests are used for comparisons between groups. The last observation carried forward (LOCF) method is used for missing values.

The number and frequency of categorical safety outcomes will be presented and compared between groups by the chi-squared test or Fisher’s exact test. The means are presented with SDs or medians with IQRs for laboratory parameters, and a one-sample *t* test is used for comparisons of the baseline and follow-up values within the group, with two-sample *t* tests used for comparisons between groups. The LOCF method is used for missing values.

Subgroup analyses for the primary and important secondary endpoints will be determined before fixing the SAP. The following pre-specified factors are used for subgroup analyses for the primary and important secondary endpoints: HbA1c (≥ 8.0%, < 8.0%), use of DPP-4 inhibitors prior to trial registration, sex, BMI (≥ 25 kg/m^2^, < 25 kg/m^2^), LDL-C (≥ 130 mg/dL, < 130 mg/dL), age (≥ 65 years, < 65 years), and the presence of treatment for existing ischemic heart disease before enrollment (PCI or CABG). The statistical significance of possible treatment effect heterogeneity between subgroups is assessed with interaction terms in repeated ANOVA. Other exploratory analyses are conducted based on the consensus of the Steering Committee. Because of the exploratory nature of these analyses for other than the primary endpoint, no correction for multiplicity is made.

All statistical analyses are performed by a study statistician (Morimoto T) and members of the data center (Institute for Clinical Effectiveness) with the use of JMP 13.1 (SAS Institute Inc., Cary, NC) and SAS 9.4 (SAS Institute Inc., Cary, NC) based on the SAP. All *P* values are two-sided, and *P* < 0.05 is considered significant other than for the non-inferiority test for HbA1c, where a one-sided *P* < 0.025 is considered significant. For baseline data, missing data are not imputed, and data with missing data are analyzed as they are. Because of the short enrollment and follow-up periods and the estimated low risk of adverse events, no interim analyses are planned.

## Discussion

This pragmatic, randomized, controlled trial was designed to investigate the comparative effects of two DPP-4 inhibitors, anagliptin and sitagliptin, on plasma levels of LDL-C in type 2 diabetic patients with a high risk for cardiovascular diseases. The lipid-lowering effect of anagliptin was first demonstrated in a phase III trial comparing anagliptin to placebo as a single agent or with concurrent anti-diabetic treatment, such as alpha-glucosidase inhibitors, biguanide, sulfonylurea, or thiazolidine in type 2 diabetic patients for 12 weeks [9]. Anagliptin significantly lowered LDL-C by 5.4 mg/dL and, though it was a before and after comparison, further reduction of LDL-C levels by 9.7 mg/dL compared to those at baseline was observed during the additional observation period extended to 52 weeks. In terms of effects of sitagliptin on lipid metabolism, a similar extended phase III trial showed no significant reduction of LDL-C [11]. Furthermore, a recent meta-analysis including 11 randomized controlled trials showed that sitagliptin alone or in combination with other drugs significantly improved levels of serum TG and HDL-C but had no effects on LDL-C levels with substantial heterogeneities [12]. Although these data suggest that LDL-C-lowering effects of anagliptin seem specific among DPP-4 inhibitors, we should, however, interpret them with great caution because measurement of lipid was not the primary end point in most studies and therefore not well standardized among studies. In addition, there has been no direct comparison between anagliptin and other DPP-4 inhibitors. Possible beneficial effects of anagliptin on lipid metabolism, therefore, should also be demonstrated in a pragmatic trial with a longer observation period that compares it to other DPP-4 inhibitor regarding LDL-C as the primary end point and enrolls more relevant patients, i.e., patients with a high cardiovascular risk with insufficient lipid-lowering despite use of statins. From these points of view, this trial was designed to enroll type 2 diabetic patients with at least one atherosclerotic vascular lesion and LDL-C levels above 100 mg/dL on statin treatment, with an observation period of 52 weeks after randomization. Given that DPP-4 inhibitors are the most frequently prescribed anti-diabetic drugs in Japan, comparing anagliptin to sitagliptin as an active control rather than placebo is more realistic and relevant to clinical practice.

## Conclusions

From the points of view discussed above, a pragmatic, unblended, randomized, controlled trial comparing the effects of anagliptin and sitagliptin on plasma LDL-C levels in type 2 diabetic patients with at least one atherosclerotic vascular lesion and insufficient LDL-C lowering despite the use of statins was designed.

## Electronic Supplementary Material

ESM 1 (DOCX 30 kb)

## Abbreviations

*LDL-C*: low-density lipoprotein cholesterol
*HbA1c*: hemoglobin A1c
*DPP-4*: dipeptidylpeptidase-4
*GLP-1*: glucagon-like peptide 1
*EDC system*: an electronic data capture system
BMI: body mass index
*TC*: total cholesterol
*HDL-C*: high density lipoprotein-cholesterol
*TG*: triglycerides
*Apo-A1*: apolipoprotein A1
*ApoB*: apolipoprotein B
*ApoE*: apolipoprotein E
*1,5-AG*: 1,5-anhydro-D-glucitol
*ApoB48*: apolipoprotein B48
*hsCRP*: high-sensitivity C-reactive protein
*IL-6*: interleukin-6
*SAP*: the statistical analysis plan
*FAS*: the full analysis set
*SAS*: the safety analysis set
*PPS*: the per protocol set
*ANOVA*: analysis of variance
*MMRM*: mixed effect models for repeated measures
*LOCF*: the last observation carried forward
*IMT*: intima media thickness

## References

[1] Beckman JA, Creager MA, Libby P (2002). Diabetes and atherosclerosis: epidemiology, pathophysiology, and management. JAMA.

[2] The emerging risk factor collaboration (2011). Diabetes mellitus, fasting glucose and risk of cause specific death. N Engl J Med.

[3] Haffner SM, Lehto S, Rönnemaa T, Pyörälä K, Laakso M (1998). Mortality from coronary heart disease in subjects with type 2 diabetes and in nondiabetic subjects with and without prior myocardial infarction. N Engl J Med.

[4] Gaede P, Vedel P, Larsen N, Jensen GV, Parving HH, Pedersen O (2003). Multifactorial intervention and cardiovascular disease in patients with type 2 diabetes. N Engl J Med.

[5] Drucker DJ (2006). The biology of incretin hormones. Cell Metab.

[6] Scirica BM, Bhatt DL, Braunwald E, Steg PG, Davidson J, Hirshberg B (2013). Saxagliptin and cardiovascular outcomes in patients with type 2 diabetes mellitus. N Engl J Med.

[7] White WB, Cannon CP, Heller SR, Nissen SE, Bergenstal RM, Bakris GL (2013). Alogliptin after acute coronary syndrome in patients with type 2 diabetes. N Engl J Med.

[8] Green JB, Bethel MA, Armstrong PW, Buse JB, Engel SS, Garg J (2015). Effect of sitagliptin on cardiovascular outcomes in type 2 diabetes. N Engl J Med.

[9] Kaku K (2012). Effects of anagliptin on serum lipids in Japanese patients with type 2 diabetes—a pooled analysis of long-term therapy with anagliptin. J Pharmacol Ther.

[10] Yano W, Inoue N, Ito S, Itou T, Yasumura M, Yoshinaka Y, et al. Mechanism of lipid-lowering action of the dipeptidyl peptidase-4 inhibitor, anagliptin, in low-density lipoprotein receptor-deficient mice. J Diabetes Investig. 2016;(2):155–60. 10.1111/jdi.12593 PMC5334308 27860391

[11] The common technical document of sitagliptin 2009 Chapter 2, 301 (in Japanese) http://www.pmda.go.jp/drugs/2009/P200900043/index.html (Accessed on10th of Jan 2018).

[12] Fan M, Li Y, Zhang S (2016). Effects of sitagliptin on lipid profiles in patients with type 2 diabetes mellitus. A meta-analysis of raaondomized clinical trials. Medicine.

