# Supplementary material for: Effect of Anagliptin and Sitagliptin on Low-Density Lipoprotein Cholesterol in Type 2 Diabetic Patients with Dyslipidemia and Cardiovascular Risk: Rationale and Study Design of the REASON Trial
Source: Cardiovasc Drugs Ther. 2018 Feb 12;32(1):73–80. doi: 10.1007/s10557-018-6776-z (PMC5843683; doi:10.1007/s10557-018-6776-z)
Supplement: Supplementary file 1 — (DOCX 30 kb) [file 10557_2018_6776_MOESM1_ESM.docx]

Supplement file

Study timeline

|  | | Pre-study Period | Baseline | Intervention Period | | | | |
| --- | --- | --- | --- | --- | --- | --- | --- | --- |
| Period | | -8～0  weeks | 0  week | 12  weeks | 24  weeks | 36  weeks | 52  weeks | discontinuous |
| Visit | | Visit 1 | Visit2 | Visit 3 | Visit 4 | Visit 5 | Visit 6 |  |
| Informed Consent | | ● |  |  |  |  |  |  |
| History Taking | | ● |  |  |  |  |  |  |
| Allocated treatment | |  |  |  |  |  |  |  |
| Adverse events | |  |  |  |  |  |  |  |
| Height | |  | ● |  |  |  |  |  |
| Waist circumference | |  | ● |  |  |  | ● | ● |
| Body weight | |  | ● |  |  |  | ● | ● |
|  | Routine blood count and biochemistry |  | ● | ● | ● | ● | ● | ● |
|  | FMD and IMT (optional) |  | ○ |  |  |  | ○ | ○ |
|  | Biomarkers for primary/secondary end points measured by central laboratory ^a^ |  | ● | ● | ● | ● | ● | ● |
|  | Biomarkers for secondary end points measured by central laboratory ^b^ |  | ● |  |  |  | ● | ● |
|  | Optional biomarkers measured by central laboratory |  | ○ |  |  | ○ | ○ | ○ |

a LDL-C、HbA1c、TC、HDL-C、TG、ApoA-I、ApoB、ApoE、Glycoalbumin、insulin、1,5-AG、C-peptide

b: Small dense LDL、ApoB48、hsCRP、IL-6、Markers for cholesterol absorption (Campesterol, Sitosterol)、 Lathosterol）、Adiponectin、Urinary albumin、Urinary creatinine、Serum for storage
